# A Technique for Improving the Precision of the Direct Measurement of Junction Temperature in Power Light-Emitting Diodes

**DOI:** 10.3390/s21093113

**Published:** 2021-04-29

**Authors:** Demetrio Iero, Massimo Merenda, Riccardo Carotenuto, Giovanni Pangallo, Sandro Rao, Gheorghe Brezeanu, Francesco G. Della Corte

**Affiliations:** 1 Department of Information Engineering, Infrastructure and Sustainable Energy (DIIES), Mediterranea University of Reggio Calabria, 89124 Reggio Calabria, Italy; massimo.merenda@unirc.it (M.M.); r.carotenuto@unirc.it (R.C.); giovanni.pangallo@unirc.it (G.P.); sandro.rao@unirc.it (S.R.); 2 HWA srl-Spin Off dell’Università Mediterranea di Reggio Calabria, Via Reggio Campi II tr. 135, 89126 Reggio Calabria, Italy; fg.dellacorte@unina.it; 3 Faculty of Electronics Telecommunications and Information Technology, University Politehnica Bucharest, 061071 Bucharest, Romania; gheorghe.brezeanu@dce.pub.ro; 4 Dipartimento di Ingegneria Elettrica e delle Tecnologie dell’Informazione (DIETI), University of Naples Federico II, Via Claudio 21, 80125 Naples, Italy

**Keywords:** junction temperature, light-emitting diode, temperature sensors

## Abstract

Extending the lifetime of power light-emitting diodes (LEDs) is achievable if proper control methods are implemented to reduce the side effects of an excessive junction temperature, *T_J_*. The accuracy of state-of-the-art LED junction temperature monitoring techniques is negatively affected by several factors, such as the use of external sensors, calibration procedures, devices aging, and technological diversity among samples with the same part number. Here, a novel method is proposed, indeed based on the well-known technique consisting in tracking the LED forward voltage drop when a fixed forward current is imposed but exploiting the voltage variation with respect to room temperature. This method, which limits the effects of sample heterogeneity, is applied to a set of ten commercial devices. The method led to an effective reduction of the measurement error, which was below 1 °C.

## 1. Introduction

Light-emitting diodes (LEDs) represent a highly efficient solid-state light source and in the last years, their use has been widespread over many market sectors, such as automotive, street lighting, architectural, and industrial, as well as commercial and residential lighting, and biomedical devices [1,2,3,4,5].

Despite the efficiency and the long-term durability claimed by LED manufacturers, high power LEDs still suffer from excessive heating, which affects their luminous flux and operating lifetime [6,7,8,9]. Proper heat dissipation systems, whether active or passive, can mitigate these side effects, but impose higher costs and more performing form factors of the lamp housing [10,11]. In order to avoid unnecessary oversizing of dissipation systems, and thus keeping costs low while preserving the lamps’ health over time, it is essential to track the exact junction temperature (*T_J_*) of LEDs. Effectively coping with this problem, which is common to many high power density solid-state devices, would benefit from the deployment of device-integrated sensors, resulting in improved robustness and enriched functionality.

Technological solutions that exploit sensorless approaches are of great interest because of the relaxation of the specifications regarding electronic design, lifetime duration, reliability, and repeatability of the transducer itself. Furthermore, indirect measurement through a separate external sensor could lead to offsets, or substantial differences, in the tracked temperature values.

Several approaches have been proposed for the indirect measurement of *T_J_*, such as those based on electroluminescence, light spectrum, Raman spectroscopy, or liquid crystal thermography [12,13,14,15]. *T_J_* can be also estimated starting from the thermally dissipated power *P_D_* and the thermal resistance *R_Th_* of the device, being in fact *T_J_ = P_D_ · R_Th_ + T_A_*, with *T_A_* being the environment temperature [16,17]. However, it is not easy to know *P_D_* and *R_Th_*, because *R_Th_* changes over time and with temperature itself [17], and only a fraction of the electric power of an LED is dissipated in the form of heat. Other sensorless ways of measuring an LED *T_J_* consist of using some temperature-dependent electrical parameters, such as the reverse saturation current [18] or the forward junction voltage *V_J_* under a constant current [19,20,21]. Recently, a detailed study based on the measurement of *V_J_* was presented in [22]. There, a set of measurements was carried out to identify the most suitable bias (or probe) current range where the LEDs can work as highly linear temperature sensors. The tests were carried out on different devices at various temperatures in steps of 10 °C in a thermostatic oven, acquiring the current-voltage (*I-V*) characteristics from 10 μA to 10 mA. The current-voltage-temperature characteristics were subsequently elaborated, and the linear fitting of the *V-T* points was calculated at each probe current, allowing to subsequently estimate the actual *T_J_* by simply measuring the forward voltage drop. To demonstrate the practical feasibility of the principle, a custom-designed, microcontroller-based circuit, fully exploiting the above recalled technique, was presented and characterized in [23,24].

In this work, that sensorless technique, based on the measurement of the junction forward voltage, is further improved to reduce the estimation error of *T_J_*, specifically when this error is due to the dispersion of the *I-V-T* characteristics among various devices, which might be large even for devices bearing the same part number. To address this issue, the new methodology, that again relies on measuring the voltage drop at the LED terminals at a fixed forward current, has been extended to include the subtraction of *I-V-T* offset curves initially measured at room temperature. The result of the characterization procedure is a linear ∆*V-T* fitting that provides current-dependent coefficients, which effectively reduces the average error. This method has been characterized and the results are discussed in comparison with the previous *T_J_* measurement procedure [22,23].

The paper is organized as follows: Section 2 summarizes the theory behind the measurement technique and outlines the novel technique; in Section 3 and Section 4, the experimental results obtained on commercial power LEDs and the relative discussion are presented, respectively. Conclusions are drawn in Section 5.

## 2. Method

Studies have already demonstrated a strong linear relationship between the voltage drop across a semiconductor junction and its temperature for several solid-state devices, provided that a constant and proper forward current is set [25,26,27,28,29,30]. In particular, the forward voltage of a P-N junction, biased at a constant current level where the diffusion component is largely predominant, is given (neglecting the impact of the series resistance) by [22,28]:

(1) $$V_{J}\left(T_{J}\right)=\frac{kT_{J}}{q}ln\frac{I_{D}}{BT_{J}^{b}}+\frac{E_{G}}{q},$$

where *T_J_* is the junction temperature, *I_D_* is the device current, *k* and *q* are the Boltzman constant and the elementary charge, respectively, and *E_G_* is the band gap energy of the semiconductor at 0 K. *B* and *b* are two constants with temperature. Using, e.g., LEDs as absolute temperature sensors over moderate domains, Equation (1) yields high sensitivities (in excess of 1.5 mV/°C, depending on the bias current levels) and reasonable linearity [19,20,21,22,31,32], such that the above relation can be written as:

(2) $$V_{J}=S\cdot T_{J}+Q,$$

where *S* (V/°C) and *Q* (V) are two coefficients that need to be found at each current, respectively representing the slope (sensor sensitivity) and the intercept of the characteristic in Equation (2). Once these two coefficients are known at a given probe current, the equation makes it possible to determine the junction temperature by measuring the forward voltage of the LED.

Equation (1) evinces two significant causes for linearity degradation and sensitivity inconsistency:

- Non-linearity in the temperature-dependence logarithmic term, which becomes significant when extending the *T* domain; and
- Fluctuations in *B* and *b* parameters, primarily due to technological heterogeneity.

Moreover, LEDs might show slight differences among their *I-V* characteristics connected with fabrication tolerances [33,34]. In view of the above, the calculated coefficients *S* and *Q* may show large standard deviations, leading to large uncertainties in the estimated *T_J_*. As it will be shown hereafter, it is possible to manage this issue.

Effects of these sources of performance degradation can in fact be mitigated by using methods based on differential forward voltage techniques [35], relying on the measurement of ∆*V_J_ = V_J_ − V_J_*_0_ with *V_J_*_0_ being the voltage drop across the LED junction at a reference junction temperature (*T_J_*_0_). In this case, the junction temperature can then be estimated from:

(3) $$\;\;\;\;\;V_{J}\left(T_{J}\right)-V_{J0}\left(T_{J0}\right)=-\frac{1}{T_{J0}}\left(\frac{E_{G}}{q}-V_{J0}\right)T_{J}+\left(\frac{E_{G}}{q}-V_{J0}\right)+b\frac{kT}{q}ln\frac{T_{J}}{T_{0}}≅-\frac{1}{T_{J0}}\left(\frac{E_{G}}{q}-V_{J0}\right)T_{J}+\left(\frac{E_{G}}{q}-V_{J0}\right),$$

which takes into account that *I_D_(T_J_) = I_D_(T_J0_) = const.* This relation can be rewritten in the form of Equation (2):

(4) $$∆V_{J}=S^{\prime \;}T_{J}+Q^{\prime }.$$

In practice, with reference to each LED of a given set, by subtracting its *I-V* characteristic at room temperature (*T_J_*_0_) from the *I-V* characteristic at the unknown temperature, a substantial reduction of the variability connected to the technological heterogeneity is observed, which allows for a more accurate determination of *T_J_* even if the same values of *S′* and *Q′* are used for all those LEDs. The adopted procedure is detailed in the following.

The *I-V* characteristics of ten LEDs with the same part number were measured at various temperatures in the range of interest, in steps of 10 °C. The tests consisted in slow cycles of temperature ramp-up and ramp-down in a thermostatic oven with a regulation precision of ±0.3 °C and temperature uniformity of ±1 °C. After setting the oven temperature, the *I-V* characteristics were acquired once the temperature fully stabilized inside the oven chamber. The devices were constantly kept off in the meantime. Once thermal equilibrium was reached, the devices, the board they are soldered on, and the oven, were at the same temperature. At this stage, the current-voltage characteristic of each LED was measured by means of an Agilent 4155C semiconductor parameter analyzer by gradually raising the forward current pulses from 10 μA to 10 mA, in steps of 10 μA. The actual temperature was measured by means of a PT100, with an accuracy of ±0.15 °C, firmly attached to the LEDs baseplate, and read with an HP 34401A digital multimeter, with a declared accuracy of ±(0.01% of reading +0.01 Ω). The same procedure was then repeated at all temperatures.

The room temperature characteristic of each LED was assumed as an offset, from which the new *I-*∆*V(I)-T* characteristics were calculated, where ∆*V(I) = V(I) − V_J0_(I)* at each current and temperature, and $V_{J0}\;$ is the forward voltage at room temperature and chosen probe current *I*. The new *I-*∆*V-T* characteristics were subsequently elaborated and the linear fitting was calculated in order to assess the degree of linearity of the ∆*V-T* responses, and to extract their best linear fit, from which the new sensitivity *S′* and intercept *Q′*, together with their standard deviations could be calculated and assumed valid for all of the devices in the set. The junction temperature of each device can then be estimated by measuring its forward voltage drop and applying Equation (4).

## 3. Results

Tests were conducted to provide evidence of the improved measurement precision over a wide span of temperature and forward current. In this study, a commercial white-light power LED (Cree XQAAWT-02-0000-00000B4E3 [36]) was considered. According to its datasheet, the maximum forward current is 300 mA, with a maximum allowed *T_J_* of 150 °C.

**Figure 1:**
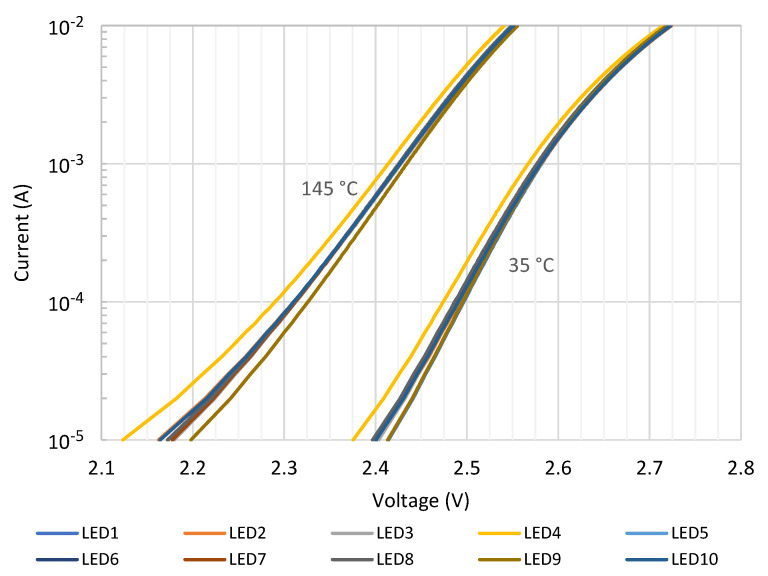
Current-voltage characteristics of ten LED samples [36] at 35 °C and 145 °C.

The tests were carried out in the temperature range from 35 °C to 145 °C. The *I-V* characteristics of the ten samples are reported in Figure 1 at the lowest and highest temperatures, clearly showing the existence of a dispersion among them. By firstly using the same technique described in [22], the values of *S* and *Q*, averaged over the ten samples, were calculated at each current together with their standard deviations. *T_J_* extraction tests were then run to assess and compare the quality of the two extraction techniques.

**Figure 2:**
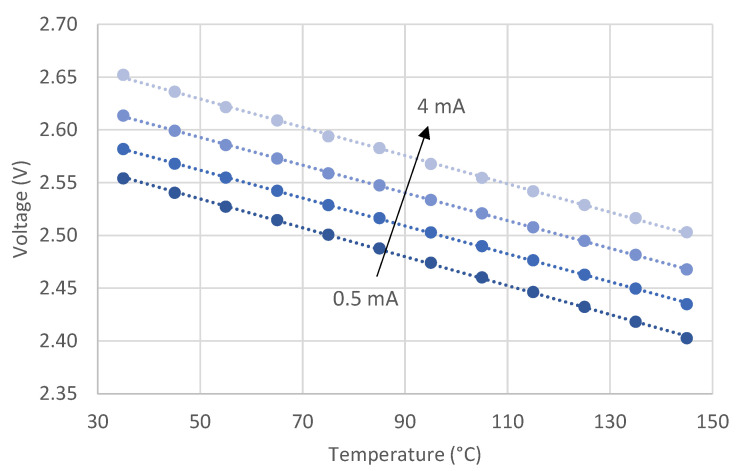
Measured (dots) forward voltage versus temperature at four increasing probe currents for one of the LEDs used in our experiments; data are fitted with their linear interpolation (lines).

As already reported in [22], and confirmed hereafter in Section 4, the average error changes with the probe current, because different probe currents correspond to different *V-T* characteristics for each LED. This is clearly shown in Figure 2, which reports an example of *V-T-I* dependence for one of the LEDs used in our experiments.

The process of selection of the optimal current takes into account the *V-T* curve linearity in the temperature range of interest and the associated average error. In our case, the lowest average error, across all LEDs, was registered at the reference current of 1.88 mA, for which the sensitivity *S* was −1.35 mV/°C and the intercept of *V-T* characteristic was *Q* = 2.65 V, with standard deviations respectively of 2.1 × 10^−5^ V/°C and 3.8 × 10^−3^ V. The intercept value was close to *E_G_*/*q* amount.

**Figure 3:**
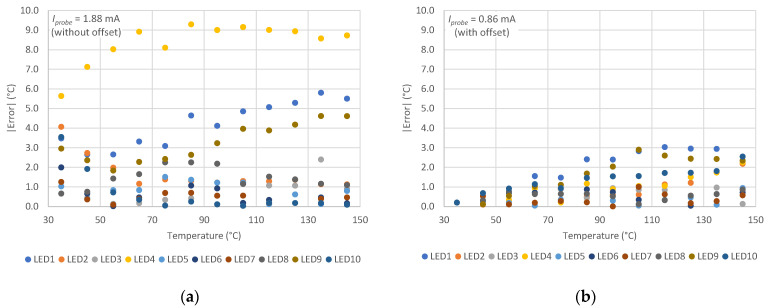
Absolute errors of measured temperature for the ten considered LEDs obtained by using the extraction technique presented in [22] (**a**), and the new calculations with offset (**b**). Measurements are run at 1.88 mA for (**a**) and 0.86 mA for (**b**), which are the probe currents, respectively, providing the lowest errors for the two methods.

The results are summarized in Figure 3a, showing that some devices provide errors well above 4 °C. This is due to the observed dispersion among the *I-V* characteristics of Figure 1. At this probe current, the average absolute error for all LEDs across the whole temperature range was 2.23 °C with a standard deviation of 2.37 °C.

Afterward, the whole temperature extraction procedure was repeated, this time using the *I-*Δ*V-T* characteristics as introduced in Section 2. As will be shown in Section 4, in this case, the lowest error was obtained at a probe current of 0.86 mA. The results for all LEDs are summarized in Figure 3b. From Figure 1, it results that at this current, the series resistance effect can be neglected. The values calculated for sensitivity and intercept of Δ*V_J_* − *T_J_* line were *S′* = −1.37 mV/°C and *Q′ =* 47.62 mV respectively, with standard deviations of 2.1 × 10^−5^ V/°C and 6.4 × 10^−4^ V, respectively. Note that from Equation (3), the following is obtained:

(5) $$\frac{Q\prime }{S\prime }=\frac{\left(\frac{E_{G}}{q}-V_{J0}\right)}{\frac{\left(\frac{E_{G}}{q}-V_{J0}\right)}{T_{J0}}}=T_{J0}.$$

Using the above calculated *Q′* and *S′* values, *Q′/S′* = 35 °C, an amount which coincides fairly well with the reference temperature (Figure 1).

This time, the average absolute error across the whole temperature range was only 0.88 °C with a standard deviation of 0.77 °C, both considerably better than those obtained with the previous methodology.

## 4. Discussion

**Figure 4:**
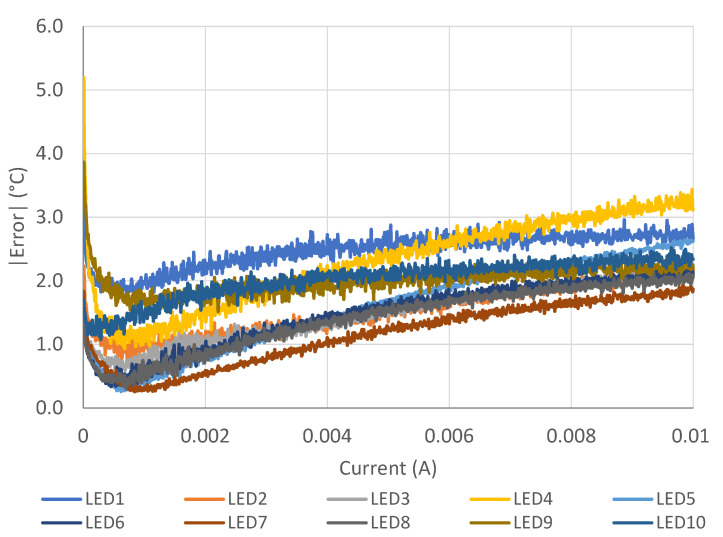
Average error at different biasing currents for the ten different LEDs, obtained by applying the offset technique described in the text.

Despite an error that increases for temperatures above 80 °C (Figure 3), the novel method provides notably lower errors over the entire temperature range. The behavioral characteristics, obtained by subtracting the *I-V* characteristics at room temperature from those at the temperature of interest, are much less dependent on the device diversities due to production process tolerances. Figure 4 represents the absolute average error obtained with the proposed method at different probe currents, for each LED used in our experiments. The graph suggests that good currents are below 2 mA.

**Figure 5:**
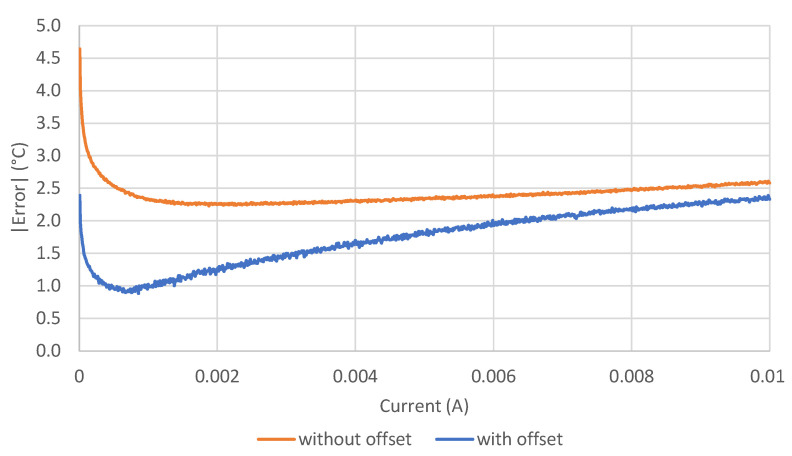
Comparison of average absolute error across all LEDs for the two methods at biasing currents varying from 10 μA to 10 mA.

Figure 5 shows the comparison of the average error across all LEDs at all currents for the two methods. With the new method, the average error remained below 1.5 °C over a wide current range. As anticipated in Section 3, the minimum error was 0.88 °C at a current of 0.86 mA. It is worth noting that at this low current, the LEDs emit a very weak radiation or they produce no emission at all.

Table 1 shows a comparison of the performances of several *T_J_* measurement techniques. The table indicates the average error across the whole temperature range, except for [21], in which the minimum peak-peak error at an ideal temperature is reported. Moreover, in [21], the LEDs were individually calibrated; when using a batch calibration in which the average coefficients for all samples are considered, errors increase significantly. It should be considered, however, that the average error also depends on the number of LEDs used in the experiments and their *I-V* characteristics dispersion, which is bound in turn to the specific LED type.

## 5. Conclusions

A method for improving the measurement accuracy of the junction temperature of LEDs, based on the tracking of the voltage across LED terminals at a known current, was proposed. Compared to previous techniques, the new procedure involves the off-setting of the measured forward voltage drop at the unknown temperature by the voltage drop at room temperature, provided the two values are measured at the same probe current. The technique is able to counteract the dispersion of *I-V* characteristics of LEDs with the same part number, making it possible to rely on just two values (*S′*, *Q′*, valid for all devices) for the extraction of the *T_J_* for each of them. For the same set of ten devices, a notable reduction of the average measurement error was in fact obtained in comparison to the standard method, which decreased from 2.23 °C to 0.88 °C.

The technique can be easily implemented with a microcontroller-based electronic circuit that imposes a current to the LED and measures the forward voltage drop. Its implementation makes it possible to extend the lifetime of the power LEDs through suitable control methods reducing the side effects of the excessive increase of the junction temperature.

## Figures and Tables

**Table 1:** Comparison among power LED T_J_ estimation techniques.

Work	Number of LEDs Used	Probe Current (mA)	Temperature Range (°C)	Error (°C)
[21]	12	2	50–130	0.99
[37]	1	10	24–40	1
[38]	1	10 to 25	33–52	0.58
[22]	5	0.6	25–135	1.41
[23]	3	8	25–135	0.91
[24]	5 (series)	2 to 10	25–135	1.7
this work	10	0.86	35–135	0.88

## Data Availability

Not applicable.
